# How Gender Identity and Treatment Progress Impact Decision-Making, Psychotherapy and Aftercare Desires of Trans Persons

**DOI:** 10.3390/jcm8050749

**Published:** 2019-05-26

**Authors:** Toby K. Mayer, Andreas Koehler, Jana Eyssel, Timo O. Nieder

**Affiliations:** Interdisciplinary Transgender Health Care Center Hamburg, Institute for Sex Research and Forensic Psychiatry, University Medical Centre Hamburg-Eppendorf, 20246 Hamburg, Germany; toby.mayer.90@gmail.com (T.K.M.); an.koehler@uke.de (A.K.); janaeyssel@web.de (J.E.)

**Keywords:** healthcare, individual treatment progress score, non-binary, patient satisfaction, transgender, transition

## Abstract

The gender identity of trans individuals influences their treatment preferences, and this in turn seems to affect their individual treatment progress. However, there has been no research which—next to the impact of gender identity on treatment desires—has also investigated the influence of treatment progress using a measure which assumes various possible transition pathways of trans persons.Therefore, an online community survey of trans people was conducted in Germany in 2015. Data were collected via an online survey from a non-clinical sample of *n* = 415 trans individuals (over half assigned female at birth), aged 16–76 (Mean (*M*) = 38.12). Almost one fifth of participants embraced non-binary or genderqueer (NBGQ) identities. Participants progressed 60.77% (standard deviation (*SD*) = 35.21) through treatment at point of data collection, as measured by the individual treatment progress score (ITPS). All participants, especially participants assigned male at birth, differed significantly in desire to participate in decision-making processes based on transition progress; individuals without treatment experience had less desire to decide treatment plans. NBGQ participants assigned male at birth in early stages of transition had significantly more desire for psychotherapy during transition than participants of the same identity in later transition stages. All participants, especially binary participants, significantly differed in desire for aftercare based on transition progress; individuals without treatment experience indicated more desire for aftercare. Results indicate health professionals should expect changing treatment desires in trans individuals at various stages of transition, particularly at treatment start, and based on gender identity.

## 1. Introduction

Trans individuals experience an incongruence between their gender identity and sex assigned at birth, which can lead to gender dysphoria—affective and cognitive discontent with one’s assigned sex [1]. This incongruence, especially paired with gender dysphoria, often leads to the desire to transition, a process which should enable trans individuals to live as their experienced gender [2], thereby reducing gender dysphoria. Medical transition involves feminizing or masculinizing the body through hormone therapy and/or surgery to align one’s body to the experienced gender, to the extent possible [1,3,4].

Trans individuals are likely to experience positive life outcomes when receiving trans-affirmative care, including medical transition [5]. Individuals further along in transition report better mental health and experience less distress [2], as measured in decreases of avoidant coping strategies used [6], rumination [7], suicidality [8], and dysphoria [9], and improvements in quality of life [10], sexual satisfaction [11,12], body image [13,14,15,16,17], and depression and/or anxiety symptoms [14,18,19,20].

Various experiences of gender and dysphoria mean not all trans individuals require all aspects of transition-related treatment [3,21,22]. For example, many transmasculine (FTM) individuals do not require genital surgery to sufficiently affirm their sense of self [21,23,24]. Non-binary and genderqueer (NBGQ) individuals in particular often experience their genders in such ways which lead to requests for ‘partial’ treatments [25] to become androgynous [26] or less interest in passing and medical transition [27,28]. It is interesting to note that in the case of NBGQ individuals, context and age appear to play additional roles in the identification with NBGQ identities beyond subjective experiences of gender and dysphoria [29]; NBGQ individuals are more likely to be assigned female at birth (AFAB) than assigned male at birth (AMAB) [21,27,30], be university-educated, and be younger than binary individuals [21].

Recent new diagnoses and understandings of gender (dysphoria) [1,4] have resulted in improvements to transition-related care, including value placed on patient satisfaction with the treatment process itself [24]. Patient satisfaction with treatment process is often used as a measure of healthcare quality; communication with doctors/nurses, timeliness of assistance, easy explanations of treatments, and thorough discharge planning are associated with improved healthcare outcomes, such as decreased hospital re-admission rates [31], emergency department use [32], and inpatient mortality [33].

Patient satisfaction with the outcomes of medical procedures (i.e., surgery) was previously employed as the sole measure of transition-related treatment success and quality [11,15], despite trans individuals focusing on treatment process characteristics when defining positive healthcare experiences [34]. Nonetheless, several studies show high levels of overall patient satisfaction with trans healthcare services [35,36,37,38], particularly from nonjudgmental, knowledgeable professionals [34,37,39].

Updates to manuals and treatment guidelines have placed much emphasis on patient self-determination [24,40], which is of current interest for patient satisfaction. This decision is supported by literature; requests for tailored treatments are increasing [21,25], and higher satisfaction is reported when treatment approaches are flexible [39] and offer control over treatment planning [34]. However, research is lacking on how patients experience their role in decision-making processes.

Requirements of extensive psychotherapy often decrease satisfaction [39,41]; however, rates of suicidality [30,42] and depression and anxiety [7,43] often remain high after transition, indicating trans individuals may benefit from ongoing mental healthcare. In many countries, such as those following the Standards of Care 7 (SoC7) [3], psychotherapy is no longer a strict prerequisite for medical transition [3,44], which can help psychotherapy to remain useful for the transition process [5,45]. With these contrasting aspects, it is important to further investigate how trans individuals perceive psychotherapy.

Due to risks, complications, and recovery processes [46,47], quality aftercare (wound care, physiotherapy, etc.) influences aesthetic and functional results of gender affirmative surgeries [48,49], which trans individuals rate as key factors for satisfaction with surgical outcomes [15,17]. As relaxed psychotherapy requirements [3,44] and recognition of NBGQ identities [1,3,4,5] mean more individuals can access surgery [3,30,50], experiences of aftercare are important to investigate.

Parallel to new diagnoses and understandings of gender, research methods in the field of trans healthcare have been updated to better reflect the diversity of trans experiences. The Individual Treatment Progress Score (ITPS) [21] is a novel new metric score to measure transition progress, which challenges previous notions that all trans individuals move through the same predefined, linear treatment pathway starting with hormones and ending in genital surgery [25,26], or that all individuals need the same number of treatments to consider their transition complete. The ITPS allows for comparison of treatment progress of individuals across genders, including NBGQ identities, or transition goals and thus better captures the social and medical transition realities of trans individuals reflected in the findings of current research [3,26,51].

The ITPS is a continuous value between 0–100% calculated for each participant, based on the number of treatments participants indicated they had completed divided by the number of treatments they still have planned [21]. It must be acknowledged that the transition end-point is potentially changing in nature when it is individually determined in this way; individuals may desire to undergo another treatment at a later date, thus changing their original ITPS value. The ITPS is also of interest for the investigation of patient satisfaction with transition-related care asresults of other studies have determined that transition progress, when measured by the ITPS, explained a significant amount of variance in quality of life and mental health outcomes in trans individuals [24,52].

Research on patient satisfaction with other treatments may not generalize to trans individuals, and the few studies investigating satisfaction with transition-related care [35,36,37,39] did not analyze differences in satisfaction throughout the transition process or based on gender identity. As changing treatment approaches cast uncertainty onto patient satisfaction with decision-making, psychotherapy, and aftercare, this study aims to use a more appropriate research tool, namely the ITPS, to close the gap in research on trans patient satisfaction with treatment by answering the following questions:

### 1.1. How Does Sex Assigned at Birth and Gender Binarity Influence Trans Individuals’Desire to Participate in Decision-Making, Psychotherapy, and Aftercare?

Trans individuals of different genders often undergo different types of transition treatments, due to the types of procedures available based on their sex assigned at birth [3] or experiences of gender and/or dysphoria [21,23,25], for example. Furthermore, trans individualsface various types of discrimination, which is often gender identity-specific [30,53,54], resulting in various levels of psychological well-being among trans individuals of certain identities [55]. These aspects may influence the way trans individuals of different gender identities (mentally) approach transitioning as well as their treatment needs, and it is thus assumed that there is a statistically significant difference in desires to participate in decision-making, psychotherapy, and aftercare between trans individuals of different gender identities (i.e., NBGQ vs. binary, AMAB vs. AFAB).

### 1.2. How Do Trans Individuals Differ in Their Desire to Participate in Decision-Making, Psychotherapy, and Aftercare at Different Stages in Their Transition, as Measured by the ITPS?

Research has shown that trans individuals’ affective and cognitive states change during the transition process. For example, trans individuals face the highest risk for mental health issues before treatment has begun [1,56], while improvements in psychopathological symptoms [43] and quality of life [57] are found towards the end of transition. Furthermore, trans individuals often have negative experiences accessing (trans) healthcare, leading to expectations of future discrimination by health professionals and forgoing care [22,54,58]. It is thus assumed that trans individuals in the early stages of transition, with potentially poorer mental health and fewer previous healthcare experiences on which to base their desires, statistically significantly differ in their desire to participate in decision-making, psychotherapy, and aftercare compared with trans individuals in later transition stages. Furthermore, it is assumed that trans individuals with no treatment experience, who have may have the poorest mental health and have treatment expectations which may not fully match reality due to lack of healthcare experiences [59], differ in their desires compared with individuals with treatment experience.

## 2. Experimental Section

### 2.1. Procedure

The researchers developed a questionnaire using a participatory research approach. They created a working group by collaborating with two trans-identified representatives from local trans support groups, as well as a psychiatrist and endocrinologist with experience providing transition-related medical care. The working group created a list of relevant topics and revised drafts of the questionnaire’s structure and items. The final version of the questionnaire covered trans-related sociodemographic data, treatment status (undergone treatment, planned treatment), treatment desires, and healthcare concerns [24].

Participants had to identify as trans/non-cisgender (e.g., transgender, genderqueer) and be at least 16 years old to be eligible for participation. Accessing transition-related healthcare or plans to transition were not required. Participants were recruited across Germany. Links to the study were emailed to trans support/activist groups and shared in trans-related social media groups, and flyers were posted in LGBT centers and trans healthcare practices.

Data were collected over two months in the summer of 2015. Participants represented a non-clinical convenience sample. No incentives were given for participation. Completion rate equaled 52.4% [24]. Participants confirmed informed consent electronically by clicking ‘yes’ to the corresponding question at the beginning of the questionnaire. The original project, which provides data for the current study, received approval from the ethics committee of the Chamber of Psychotherapists Hamburg (approval number 05/2015-PTK-HH).

### 2.2. Participant Demographics

The sample consisted of *n* = 415 trans individuals, aged 16–76 (*M* = 38.12, *SD* = 12.82). Regarding sex, *n* = 216 (52.0%) and *n* = 199 (48.0%) participants were assigned female and male at birth, respectively. Of the participants, *n* = 339 (81.7%) individuals had a binary gender identity and *n* = 76 (18.3%) individuals had a NBGQ identity. Of the AFAB participants, 48.1% and 69.7% embracedbinary and NBGQ identities, respectively [21].

Most participants reported experience with transition-related treatments; *n* = 304 (73.3%) participants reported having undergone at least one somatic procedure. At the time of data collection, participants were 60.77% (Median = 66.67, *SD* = 35.21) finished with their transitions, as measured by their ITPS [21].

### 2.3. Measures and Operationalization

Desire to participate in decision-making, psychotherapy, and aftercare comprised the dependent variables (ten other treatment desires were analyzed but did not produce significant results). ‘Desire for psychotherapy’ was measured via the item “In my view, it is helpful to receive psychotherapeutic counselling during the transition process” with ‘Yes/No/Unsure’ as response options. ‘Desire for aftercare’ was measured via an item “In my opinion, it is helpful to be able to access rehabilitation offers after my surgical treatment(s) as a day patient or inpatient” with responses on a 6-point Likert scale, ranging from 1 = strongly disagree to 6 = strongly agree.

‘Desire to participate in decision-making’ was measured via eight questionnaire items using the same 6-point Likert scale. Two items (“I want to decide how much I am going to be involved in decisions for or against specific treatment options”; “I want the healthcare professionals and myself to share responsibility in deciding which treatment might be best for me”) were not included in the operationalization as they did not clearly reflect preference for involvement. The creation of a mean score by combining responses to the six items proved unsuccessful as reliability analysis indicated an unacceptable level of internal consistency (α = 0.18). As such, a different score was calculated for each participant from the sum of their responses to three items desiring high decision-making power (“I know exactly which treatments I want to undergo and just want the respective healthcare professional to implement them as quick as possible”; “Ultimately I want to decide on my medical treatment, after having looked in great depth at the opinion of the healthcare professional involved”; “I want to decide which medical treatment I undergo (e.g., hormone treatment, genital surgery)”) minus the sum of their responses to three items desiring low decision-making power (“Sometimes it is convenient to just let go of all responsibility during medical treatment”; “I want the healthcare professional involved in my treatment to make the ultimate decision on my medical treatment, after having consulted me on my opinion”; “I want to leave all decisions regarding my medical treatment to the healthcare professional involved in my treatment”) to operationalize the variable ‘desire to participate in decision-making’. A value less than zero indicates a preference for low/no decision-making power, while a value greater than zero indicates a preference for high decision-making power [24].

Transition progress comprised the independent variable, measured by the ITPS. As previously mentioned, the ITPS is a metric score with a continuous value between 0–100% calculated for each participant, based on the number of treatments participants indicated they had completed divided by the number of treatments they still have planned on corresponding questionnaire items which listed various possible transition-related treatments and provided a space to indicate other treatments not otherwise listed [21]. For example, a transfeminine participant may indicate that she plans to undergo hormone therapy, hair removal, facial feminization surgery, and genital surgery to complete her transition but at the time of data collection had only undergone hormone therapy. This individual would thus have an ITPS value of 25%.

Responses to the item “What is your gender assigned at birth?” were used for the data for sex assigned at birth. Gender binarity was operationalized as having either a binary or NBGQ identity. Participants who embracedat least one NBGQ term on the questionnaire, or who wrote in their own NBGQ identity, were assigned to the NBGQ category [21].

The different stages in transition were operationalized by dividing participants into groups based on ITPS: Participants were in the ‘early stages’ of transition with an ITPS below 50% and in the ‘later stages’ with an ITPS of 51% or above. In the aforementioned example with the transfeminine participant, her ITPS value of 25% would put her in the ‘early stage’ of transition according to this operationalization. Based on individuals who undergo a ‘complete’ transition (although this in not assumed with the ITPS), there is a potential change in nature of treatments at this 50% mark which may influence treatment desires. Earlier stages are often marked by psychotherapy, during which many individuals do not feel like transition has truly begun [35], hormones, or hair removal. Later stages are more likely to be marked by invasive surgical interventions, such as breast/chest or genital surgery [3], after which individuals often first feel like their experienced gender [17] or report sufficiently reduced dysphoria [15,22], but also deal with postoperative pain and/or depression [60], complications, and side effects [47,61,62]. Participants with an ITPS of 0% were assigned to the ‘no treatment stage’ of transition group.

### 2.4. Data Analysis

Results of the Shapiro–Wilk test of normality indicated that the data split at 50% ITPS were not normally distributed (*W* = 0.71, *p* = < 0.001), as approximately 30% of the participants had completed their transition and thus had an ITPS value of 100%. As such, Kruskal–Wallis H tests were conducted to analyze in-between subject group differences for variables ‘desire to participate in decision-making’ and ‘desire for aftercare’, for the three groups of transition stages: No treatment, early, and late stages. Post hoc pairwise comparisons using Dunn’s (1964) procedure with Bonferroni adjustment were then performed to determine which group(s) differed from one another. Missing values were excluded on a test-by-test basis from analysis. Adjusted alpha level after the Bonferroni correction was 0.005.

Chi-square tests of homogeneity were conducted to analyze group differences for the variable ‘desire for psychotherapy’. Fisher’s exact tests were calculated when expected cell counts were not greater than five. To conduct 2 × 2 tests, participants who responded ‘No’ or ‘Cannot/do not want to answer’ were combined into one group and compared against participants who responded ‘Yes’. Participants with an ITPS of 0% and an ITPS of 1–50% were combined into one group and compared against participants with an ofITPS 51–100%. Adjusted alpha level after the Bonferroni correction was set to 0.025.

Analyses were run for the sample as a whole, as well as the sample split by sex assigned at birth (AMAB vs. AFAB), gender binarity (binary vs. NBGQ), as well as sex assigned at birth *and* gender binarity (binary AMAB vs. binary AFAB vs. NBGQ AFAB vs. NBGQ AMAB). SPSS 20.0 [63] was used for all calculations.

## 3. Results

### 3.1. Descriptive Results

Scores for the variable ‘desire to participate in decision-making’ were negatively skewed in the sample (*n* = 414), with a mean score of 8.58 (*SD* = 4.45, 95% CI (8.15, 9.01)) from a range of possible scores from −6 to 15. Scores for the variable ‘desire for aftercare’ were similarly negatively skewed in the sample (*n* = 407). The mean ‘desire for aftercare’ score was 4.30 (*SD* = 1.26, 95% CI (4.18, 4.42)) from a range of 1 to 6). Regarding response frequencies (*n* = 414) for the variable ‘desire for psychotherapy’, 311 participants (75.1%) answered ‘yes’, indicating they would find it helpful to receive psychotherapy during transition, while 58 participants (14.0%) answered ‘no’, and 45 (10.9%) did not want to/could not answer the question. These results indicate participants highly desired participation in decision-making processes, psychotherapy, and aftercare.

### 3.2. Inferential Results

Results of the Kruskal–Wallis H test determined that there were statistically significant differences in scores for the total sample and the AMAB participants for the variable ‘desire to participate in decision-making’ (see Table 1). Subsequent post hoc analysis revealed statistically significant differences between the 0% ITPS and 51–100% ITPS groups for both the total sample and AMAB participants (see Table 2).

These results indicate that for both the total sample, as well as the AMAB participants, individuals with no treatment experience (ITPS of 0%) desired significantly less involvement in decision-making processes (i.e., approve of healthcare professionals having more say in decisions) during their transition than individuals in the later stages of transition.

Since the 0% ITPS group only differed from the 51–100% ITPS group in this desire, the results only partially confirm the assumption in the second research question, which suggested trans individuals in the three transition stages would significantly differ from each other in their treatment desires. Furthermore, the fact that only the results of the AMAB participants remained statistically significant once the analysis was applied to different gender identities indicates that for all other identities, sex assigned at birth influenced participants’ need for involvement in decision-making processes and not just their stage in transition. Thus, the assumption in the first research question, which stated trans individuals of different gender identities would have different treatment desires, is also supported.

Results of the Kruskal–Wallis H test determined that there were statistically significant differences in scores for the total sample and the binary participants for the variable ‘desire for aftercare’ (See Table 3). Subsequent post hoc analysis revealed statistically significant differences between the 0% ITPS and 51–100% ITPS groups for the total sample and binary participants (See Table 2).

These results indicated that for the total sample and the binary participants, individuals with no treatment experience (ITPS of 0%) had a significantly stronger desire for aftercare than individuals only in the later stages of transition (ITPS between 51–100%).

Since the 0% ITPS group only differed from the 51–100% ITPS group in this desire, these results again only partially confirm the assumption of research question 2, which suggested trans individuals in all three treatment stages would significantly differ from one another in their treatment desires. Furthermore, the fact that only the results of the binary participants remained statistically significant once the analysis was applied to different gender identities indicates that for all other identities, gender binarity and not just stage in transition influenced participants’ desire for aftercare. Thus, the first assumption, which stated that trans individuals of different gender identities would have different treatment desires, is once again supported.

Results of the Fisher’s exact test determined that only the two groups of NBGQ AMAB participants statistically significantly differed in their desire for psychotherapy. Of the NBGQ AMAB participants who responded ‘yes’, indicating they find psychotherapy during transition helpful, 11 (84.6%) had an ITPS under 50%, while only two (15.5%) had an ITPS over 50%. By contrast, of participants who responded ‘no’, indicating psychotherapy is not helpful, only three (30%) has an ITPS under 50%, while seven (70%) had an ITPS over 50% (see Table 4).

These results indicated that being in the early stages of transition (i.e., having an ITPS under 50%) is associated with greater desire for psychotherapy, while being in the later stages (i.e., ITPS over 50%) is associated with greater disapproval of psychotherapy, supporting the assumption made in research question 2. Additionally, the fact that only the results of the NBGQ AMAB participants were statistically significant indicates that for all other identities, sex assigned at birth and gender binarity influenced participants’ desire for psychotherapy. This finding supports the first assumption, which stated trans individuals of different gender identities would have different treatment desires.

## 4. Discussion

### 4.1. Interpretation of Results

Results confirm the assumption made for the first research question, as many of the statistically significant results for the sample disappeared when analyses were conducted with sex assigned at birth and gender binarity controlled for as covariates.The nature of transition differs for NBGQ vs. binary individuals;a previous study analyzing the data found that NBGQ participants in the sample reported undergoing and planning less treatments for primary sex characteristics than binary individuals [21]. This is in line with other findings on NBGQ individuals’ treatment goals and processes [25,26,27]. Less treatment required could lead NBGQ individuals to have different treatment desires. The NBGQ participants were also younger and likely to be students, mirroring results of other studies [27,30]. Certain life experiences or maturity levels which are more likely to be experienced by NBGQ individuals, as compared to binary individuals who tend to be a more heterogeneous group, could also influence desires.

As NBGQ identities are seldom recognized legally and have only recently been addressed in scientific literature [5,26], including diagnostic manuals [1,3], NBGQ individuals face additional difficulties in comparison to binary individuals when accessing treatment or having their identities recognized from health professionals. For example, only 11% of NBGQ individuals in one study [30] received a diagnosis of gender dysphoria, without which medical transition is often not possible. As such, NBGQ individuals may forgo/be denied care or present for treatment with a binary gender presentation [22,64]. Pushes to provide treatments without the requirement of a diagnosis [3,65], for example, via the informed consent model used in many American healthcare centers [66], are gaining traction and may address some of the issues with treatment access that NBGQ individuals face. Nonetheless, this difficulty accessing care may also affect NBGQ participants’ transition-related treatment desires.

The nature of transition differs for AMAB vs. AFAB individuals as well. The AFAB individuals in the sample, regardless of binarity, were less likely to have undergone genital reconstruction surgery (10.8% phalloplasty) in comparison to AMAB individuals (45.8% vaginoplasty), similar to findings of other studies [22,30,51,67]. AFAB and AMAB individuals may thus have different transition goals, needs, or experiences on which they base their treatment desires.

All participants highly desired participation in decision-making processes during transition. This finding supports recommendations in the SoC7 that professionals work together with trans individuals to plan treatments [3] and is consistent with the findings of other studies. For example, trans individuals requested that psychotherapists respect patient autonomy and share responsibility when making decisions [68], while others desired high involvement in decision-making, via self-management of healthcare plans. Self-management increased patient satisfaction with care, as individuals could ascertain treatments best reflected needs and goals [34].

However, for the total sample, and in particular the AMAB participants, individuals without treatment experience desired significantly less involvement in decision-making than participants in the later stages of transition, supporting the assumptions made for the second research question. Trans individuals often report having to educate medical professionals on their healthcare needs when attempting to access treatments [34,51,64], indicating that patient participation in decision-making during treatment is often necessary to receive care. Trans individuals without treatment experience may be unaware of this reality and may underestimate their desired level of participation in decision-making, compared to those in later transition stages whose healthcare experiences have led to realizations of the necessity of being involved in decision-making. The fact that AMAB participants planned more treatments and were more likely to undergo genital surgery than the AFAB participants may explain why this finding occurred only in the AMAB participants; these participants in particular may have more contact with healthcare professionals for which they want to be involved, especially plans for important genital surgeries.

The majority of participants, regardless of transition progress or gender identity, desired psychotherapy, corroborating findings from other studies [35,36,38]. This finding is important, as mandatory psychotherapy and diagnoses have been heavily criticized [45,69,70]. It appears that participants in the sample are aware of psychotherapy’s benefits [3,5].

However, NBGQ AMAB participants differed significantly in their psychotherapy desires based on transition stage, thus supporting the assumption made for research question 2. The majority of these individuals in the first half of transition desired psychotherapy, while the majority in later stages did not. NBGQ AMAB individuals may have a difficult therapy process in comparison to all other genders, as therapists may be less likely to take NBGQ individuals’ experiences of gender and/or dysphoria seriously [30] or expect only AFAB individuals to identify with NBGQ genders [21,27,30]. As such, NBGQ AMAB participants in later stages of transition, who are more likely to have finished psychotherapy, may desire psychotherapy less after previous negative experiences.

In general, all participants desired aftercare, regardless of transition stage, echoing results of another study in which majority of participants requested insurance reimburse costs for rehabilitation and physiotherapy [68].

All participants, but especially binary individuals, without treatment experience had significantly stronger desires for aftercare than participants in later stages of transition, once again supporting the assumption made for research question 2. Due to reliance on third-hand information about transitioning and medical procedures [34,71,72], resulting in unrealistic expectations [59], individuals without treatment experience may overestimate the difficulty of the recovery process and more strongly desire aftercare [22]. By contrast, individuals in later stages of transition often have experience with somatic procedures and can more accurately assess (or reflect on) their post-operative needs. Furthermore, these individuals have had more experience with trans healthcare in general. Myriad studies have highlighted high frequencies of discrimination experiences and poor care [30,53,54,58], which lead to expectations of future discrimination [22,58]. As such, trans individuals in later transition stages may disapprove more of aftercare due to expectations that experiences will be negative and may choose to forgo care. Additionally, the fact that binary participants were more likely to undergo surgical treatments can explain why aftercare desires were more relevant for this group compared to the NBGQ participants.

### 4.2. Clinical Implications

The results are valuable for clinical practice as they confirm there is no one right way to provide transition-related treatments. Professionals working in transgender healthcare should expect differences in treatment desires, depending on the individual’s gender identity and transition progress, among other factors. Professionals should respond to individuals both individually and flexibly and should continuously check in with them to ensure satisfaction with treatment choices throughout transition. Healthcare professionals should encourage individuals at the start of transition to take an active role in decision-making processes [66], while those in later transition stages may need to be encouraged to make use of the opportunity psychotherapy provides for reflection through all stages of transitioning [3]. Professionals should be supportive if individuals later change their minds about previously agreed-upon aftercare plans. Flexible treatment options bookable on short notice, such as home nursing care after surgery, would be advantageous. To counter issues, providing information about transitioning/treatments, as well as a contact partner to answer questions, especially at treatment start, would be useful.

Results of the study also confirm that the ITPS is a useful way to measure transition progress across genders for trans individuals with varying planned and undergone treatments. Not only should its use be encouraged within the field of trans research, but healthcare professionals could utilize the ITPS to help clients determine their transition needs and plan their individualized treatment pathway. This could be especially useful for psychotherapists who accompany individuals throughout their transition to organize sessions.

### 4.3. Limitations

Although no data on ethnicity were collected, 90.8% of participants were born in Germany, and it can be assumed that most individuals were white. Furthermore, the majority of participants (61.9%) were atheist. The sample may thus not be representative of immigrant/ethnic minority trans individuals in Germany or other non-Western countries. Potentially due to the study’s use of an online survey, only 20.7% of participants in the current study were aged 50 or over, and the majority (62.7%) began transition within the last five years. This is nevertheless in line with other studies with majority young or recently transitioned participants [30,54]. Experiences of young/newly out trans individuals may not generalize to the needs of older generations, who may have to deal with the stress of coming out to a spouse or child(ren) [30] or experienced older, pathologizing treatment approaches [73].

Non-normal distribution of participants’ ITPS values forced use of nonparametric tests. Low numbers of participants in some groups may have affected tests’ statistical power. The 2 × 2 Chi-square tests of homogeneity/Fisher’s exact test meant participants with an ITPS of 0% were not analyzed as their own group, and the second hypothesis could not be fully tested for participants’ psychotherapy desires. Hypotheses were heavily based on discrimination experiences; however, no data were collected on this issue.

### 4.4. Future Research Directions

Future research should strive for a larger, more diverse sample to allow for participants with an ITPS of 100% to form their own group for separate analysis. This would enable investigation of individuals who have completed transition (sometimes many years ago) and determine if individuals retrospectively reporting treatment desires differ from individuals currently undergoing treatment.

The results of the current study also indicate that a longitudinal study would be advantageous and a logical next step for research in treatment desires and patient satisfaction with treatment. The same individuals should be followed throughout transition and treatment desires captured when a particular ITPS is reached. This would allow causal inferences to be made about why/when treatment desires might change and determine if treatment adherence, satisfaction, and well-being of trans individuals is affected if there are differences between desired and received treatments.

## 5. Conclusions

This study builds on the findings of previous research on patient satisfaction with trans healthcare [34,35,37,39] by highlighting the changing nature of trans individuals’ treatment desires through the transition progress. It also adds to the research on gender identity-based differences among trans individuals by exploring how the experiences of NBGQ vs. binary and AMAB vs. AFAB individuals influence treatment desires. Participants, especially AMAB individuals, without treatment experience desired less participation in the decision-making process, while NBGQ AMAB participants in early transition stages more strongly desired psychotherapy. All participants, especially binary individuals, without treatment experience more strongly desired aftercare. Thus, the assumptions made for both research questions, which suggested individuals differ in desires based on gender identity and transition progress, were supported. These findings can be used to improve patient satisfaction with healthcare by expecting, and adapting to, changes in treatment desires. The use of the ITPS to explore desires across genders and transition pathways highlights the need to offer individualized treatments with no pre-assumed endpoint.

## Figures and Tables

**Table 1:** Results of the Kruskal–Wallis H tests for the variable ‘desire to participate in decision-making’.

Sample Split	ITPS % Group	*n*	Score Distr.	Median Score/ Mean Rank ^a^	χ^2^	*p*-Value
Total	0	64	similar	7.00	10.42	**0.005**
	1–50	125		9.00		
	51–100	222		10.00		
AMAB	0	42	similar	5.50	15.96	**<0.001**
	1–50	49		9.00		
	51–100	105		10.00		
AFAB	0	22	similar	10.00	0.26	0.878
	1–50	76		10.00		
	51–100	117		10.00		
Binary	0	42	similar	7.00	9.40	0.009
	1–50	106		9.50		
	51–100	187		10.00		
NBGQ	0	22	similar	8.00	4.18	0.124
	1–50	19		8.00		
	51–100	35		11.00		
Binary AMAB	0	31	dissimilar	79.09 ^a^	0.43	0.805
	1–50	46		83.97 ^a^		
	51–100	96		80.16 ^a^		
Binary AFAB	0	11	similar	8.00	0.27	0.873
	1–50	60		10.00		
	51–100	91		10.00		
NBGQ AMAB	0	11	dissimilar	8.00 ^a^	8.93	0.012
	1–50	3		11.50 ^a^		
	51–100	9		17.06 ^a^		
NBGQ AFAB	0	11	similar	10.00	9.85	0.007
	1–50	16		10.50		
	51–100	26		9.00		

AMAB, assigned male at birth; AFAB, assigned female at birth; NBGQ, non-binary or genderqueer; a: mean rank score calculated for group instead of median.

**Table 2:** Post-hoc analysis pairwise comparisons for significant results of Kruskal–Wallis H tests.

Variable	Sample Split	Pairwise Comparison (ITPS % Group)	*p*-Value
Decision-making	Total	0 vs. 1–50	0.036
		0 vs. 51–100	**0.004**
		1–50 vs. 51–100	1.000
	AMAB	0 vs. 1–50	0.023
		0 vs. 51–100	**<0.001**
		1–50 vs. 51–100	1.000
Aftercare	Total	0 vs. 1–50	0.377
		0 vs. 51–100	**0.003**
		1–50 vs. 51–100	0.122
	Binary	0 vs. 1–50	0.043
		0 vs. 51–100	**0.002**
		1–50 vs. 51–100	0.787

Adjusted *p*-values for the post hoc analyses are presented.

**Table 3:** Results of the Kruskal–Wallis H tests for the variable ‘desire for aftercare’.

Sample Split	ITPS % Group	*n*	Score Distr.	Median Score/ Mean Rank ^a^	χ^2^	*p*-Value
Total	0	63	similar	5.00	11.98	**0.003**
	1–50	125		4.00		
	51–100	219		4.00		
AMAB	0	42	dissimilar	113.14 ^a^	9.86	0.007
	1–50	49		110.32 ^a^		
	51–100	105		87.13 ^a^		
AFAB	0	21	dissimilar	128.50 ^a^	3.78	0.151
	1–50	76		106.26 ^a^		
	51–100	114		101.68 ^a^		
Binary	0	42	similar	5.00	11.71	**0.003**
	1–50	106		4.00		
	51–100	187		4.00		
NBGQ	0	21	dissimilar	37.79 ^a^	6.87	0.032
	1–50	19		45.47 ^a^		
	51–100	32		30.33 ^a^		
Binary AMAB	0	31	dissimilar	103.52 ^a^	2.68	0.262
	1–50	46		95.46 ^a^		
	51–100	96		78.61 ^a^		
Binary AFAB	0	11	dissimilar	112.32 ^a^	6.13	0.047
	1–50	60		77.08 ^a^		
	51–100	90		79.79 ^a^		
NBGQ AMAB	0	11	dissimilar	13.64 ^a^	7.90	0.019
	1–50	3		16.17 ^a^		
	51–100	8		6.81 ^a^		
NBGQ AFAB	0	10	dissimilar	25.25 ^a^	7.85	0.020
	1–50	16		30.03 ^a^		
	51–100	24		22.58 ^a^		

a: mean rank score calculated for group instead of median.

**Table 4:** Results of the chi-square tests of homogeneity/Fisher’s exact test for variable ‘desire for psychotherapy’.

Sample Split	*n*	ITPS % Group	Response		*χ* ^2^	*p*-Value
			Yes: *n* (%)	No: *n* (%)		
Total	414	0–50	145 (46.6)	46 (44.7)	0.12	0.729
		51–100	166 (53.4)	57 (55.3)		
AMAB	199	0–50	73 (48.3)	20 (41.7)	0.65	0.419
		51–100	78 (51.7)	28 (58.3)		
AFAB	215	0–50	72 (45.0)	26 (47.3)	0.09	0.770
		51–100	88 (55.0)	29 (52.7)		
Binary	339	0–50	120 (45.3)	31 (41.9)	0.27	0.604
		51–100	145 (54.7)	43 (58.1)		
NBGQ	75	0–50	25 (54.3)	15 (51.7)	0.05	0.824
		51–100	21 (45.7)	14 (48.3)		
Binary AMAB	176	0–50	62 (44.9)	17 (44.7)	0.00	0.983
		51–100	76 (55.1)	21 (55.3)		
Binary AFAB	163	0–50	58 (45.7)	14 (38.9)	0.52	0.470
		51–100	69 (54.3)	22 (61.1)		
NBGQ AMAB	23	0–50	11 (84.6)	3(30.0)	0.56	**0.013**
		51–100	2 (15.5)	7 (70.0)		
NBGQ AFAB	52	0–50	14 (42.4)	12 (63.2)	2.07	0.150
		51–100	19 (57.6)	7 (36.8)		
